# Faster Healing and a Lower Rate of Recurrence of Venous Ulcers Treated With Intermittent Pneumatic Compression: Results of a Randomized Controlled Trial

**Published:** 2020-06-05

**Authors:** Oscar M. Alvarez, Lee Markowitz, Rachelle Parker, Martin E. Wendelken

**Affiliations:** ^a^Vascular and Wound Care Center, University Hospital, Rutgers, New Jersey Medical School, Newark; ^b^Center for Curative and Palliative Wound Care, Calvary Hospital, Bronx, NY

**Keywords:** venous ulceration, secondary lymphedema, chronic venous insufficiency, randomized controlled clinical trial, intermittent pneumatic compression

## Abstract

**Objective:** Fifty-two subjects with chronic venous insufficiency and hard-to-heal lower leg ulceration (>1-year-old and >20-cm^2^ surface area) were treated with either intermittent, gradient, pneumatic compression (n = 27) plus standard compression therapy or compression therapy alone (control). **Methods:** Compression therapy consisted of a nonadherent primary wound dressing plus a 4-layer compression bandage (n = 25). The mean age and size of the ulcers were 1.4 years and 31 cm^2^, respectively, and did not differ significantly between groups. Intermittent pneumatic compression was performed using a 4-chamber pneumatic leg sleeve and gradient, sequential pump. All pumps were calibrated to a pressure setting of 50 mm Hg on each subject, and treatments were for 1 hour twice daily. Evaluations were performed weekly to measure edema, local pain, granulation, and wound healing. **Results:** The median time to wound closure by 9 months was 141 days for the intermittent pneumatic compression–treated group and 211 days for the control group (*P* = .031). The rate of healing was 0.8 ± 0.4 mm/d for the control group and 2.1 ± 0.8 mm/d for the group treated with intermittent pneumatic compression (*P* < .05). When compared with subjects treated with standard care, the group treated with intermittent pneumatic compression reported less pain at each evaluation point for the first 6 weeks of the trial. At weeks 1, 2, and 3, the visual analog pain scores were significantly lower for the intermittent pneumatic compression–treated group (*P* < .05). **Conclusion:** These results suggest that intermittent pneumatic compression is a valuable adjunct to compression therapy in the management of large or painful venous ulcers.

Venous leg ulcers (VLUs) are the most common type of leg ulcers. VLUs, caused by chronic venous insufficiency (CVI) and venous hypertension, affect approximately 1% of the population and 3% of people older than 80 years.^1^ VLUs most often occur in the gaiter region of the lower leg, from just below the ankle up to mid-calf. In the United States, more than 7 million people have CVI.^1^^,^^2^ Normally, calf muscle contraction promotes venous return by squeezing blood in the deep veins; this pressure is prevented from reaching the superficial circulatory system by one-way valves within the perforating veins. In patients with CVI, venous pressure builds up in the superficial veins and is transmitted to the capillaries of the skin.^3^ In most cases, this venous incompetence is secondary to thrombophilia, which often damages valves.^4^ Three hypotheses have been proposed to explain how venous insufficiency leads to ulceration:

1. The fibrin cuff theory proposes that fibrin gets excessively deposited around capillary beds, leading to elevated intravascular pressure. This causes enlargement of endothelial pores, resulting in further increased fibrinogen deposition in the interstitium. The “fibrin cuff,” which surrounds the capillaries in the dermis, decreases oxygen permeability 20-fold. This permeability barrier inhibits diffusion of oxygen and other nutrients, leading to tissue hypoxia causing impaired wound healing.^5^^,^^6^
2. The inflammatory trap theory, which proposes that various growth factors and inflammatory cells that get trapped in the fibrin cuff, promote severe uncontrolled inflammation in surrounding tissue, preventing proper regeneration of wounds.^7^
3. The white cell entrapment hypothesis proposes that leukocytes trapped in the diseased vessels by reduced shear stress become activated on the endothelial surface. These leukocytes then release inflammatory mediators, leading to tissue destruction and blockage of small capillaries causing localized tissue ischemia.^8^

The hallmark to the diagnosis of CVI is hemosiderosis (staining of the skin from leaking red blood cells) in the gaiter area of the leg. The presence of microvericosoities (along the medial or lateral aspects of the mid-foot), interstitial edema, lipodermatosclerosis, stasis dermatitis, and superficial skin erosion combined with burning pain is another criterion that helps identify CVI. Frequently, patients develop CVI as a result of decreased ambulation or a gait abnormality. Therefore, they are more common with increasing age or in those with arthritis or other musculoskeletal conditions affecting normal gait.^9^ Although venous ulcers are not generally prone to acute infection,^10^ long-standing, untreated CVI can lead to secondary lymphedema and increased risk of cellulitis.^11^ In a recent study^11^ of 440 patients with lower extremity lymphedema, the most common cause was CVI or phlebolymphedema (41.8%).

The cornerstone of treatment of VLUs is compression therapy. Compression forces the fluid that has leaked into the perivascular space back into circulation. Ideal compression pressures for these patients remain unknown.

Intermittent pneumatic compression (IPC) devices have also been proven effective, but compliance and reimbursement (especially for Medicaid patients) are major hurdles. A distinct advantage of IPC therapy is that it can be done by the patient or other family member in the home with little or no training. Compression alone with multilayer or short stretch bandage systems is helpful but requires application by a skilled (trained) nurse or technician; a dedicated family member may be trained to appropriately apply the multilayer short stretch bandage system.

Venous ulcers in patients with secondary lymphedema pose a significant challenge, as these wounds are one of the most difficult to heal. These patients have significant brawny edema, fibrosis, and very large legs, making bandaging difficult as gradient compression pressures are often not achieved. IPC has been shown to accelerate the healing of venous ulcers in several randomized trials.^12^^-^16 However, it has never been shown to be more effective than standard compression provided with short stretch or multilayered bandage systems.^13^ Our goal was to investigate whether intermittent compression (IPC) assisted the healing of venous ulcers in patients with lymphedema who were already receiving standard compression with short stretch or multilayered compression therapy.

## MATERIALS AND METHODS

### Study objective and ethics statement

The purpose of this study was to assess the safety and effectiveness of IPC plus standard of care static compression versus standard of care static compression alone for the treatment of chronic VLUs. This study was conducted in accordance with the principles of Good Clinical Practice and the Declaration of Helsinki (2013), Title 21, Parts 50, 54, 56 of the US Code of Federal Regulations. Study stopping rules included stopping the study at the discretion of the principal investigator if subject safety was of concern or at the subject's request.

The study protocol, investigators, and consent documents were reviewed and approved by an accredited institutional review board (IRB) (Calvary Hospital IRB, Bronx, NY), and all patients provided written informed consent before participating in the study. This study was registered at ClinicalTrials.gov as NCT02750280.

### Study design and study population

The study was a prospective, randomized-controlled, parallel-group, comparative trial. Eligible subjects aged 18 to 85 years were randomly assigned to receive either control treatment, consisting of multilayer compression bandage therapy alone or IPC therapy plus compression bandaging. Subjects were followed up to 12 months for analysis of safety and efficacy. Endpoints were prospectively set at 8 months. Subjects were entered into the study after informed consent was obtained. Those who qualified were assigned to either the IPC or control treatment group according to a computer-generated randomization schedule. A total of 65 subjects were screened and 52 subjects were treated, with 27 randomized to compression bandage therapy alone (control) and 25 to IPC therapy. The ulcers were secondary to venous insufficiency, had to be open for a minimum of 1 year, had to be larger than 20 cm^2^, and the degree of local wound pain had to be more than 6 on a visual analog scale (VAS) of 0 to 10. Significant arterial insufficiency had to be excluded by demonstrating an ankle-brachial index of more than 0.75. Exclusion criteria included active infection, ulcers of nonvenous etiology, current use of systemic corticosteroids, chemo- or radiotherapy, confinement to bed or chair, and active participation in another investigational study.

### Treatment protocol and follow-up

**Figure 1:**
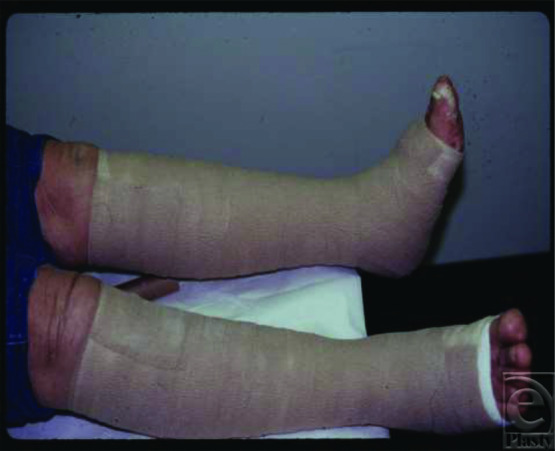
Compression bandaging using the 4-layer bandage system.

**Figure 2:**
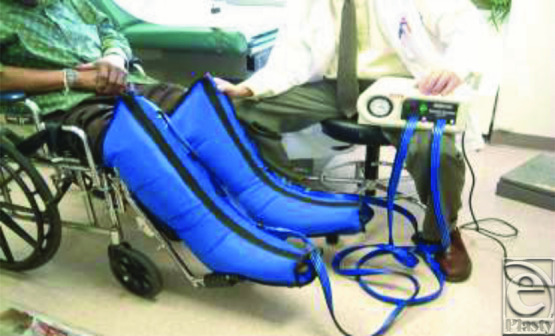
IPC therapy with intermittent, pneumatic, gradient, sequential compression pump and sleeves. IPC indicates intermittent pneumatic compression.

The ulcers of the control subjects were dressed and bandaged using the Profore 4-layer bandage system (4-LB; Smith and Nephew, Largo Fla; Fig 1). In the IPC treatment group, the ulcers were dressed and bandaged using the same 4-layer bandage system described for the control group. In the IPC group, additional compression therapy was provided by a 4-chamber intermittent gradient, sequential, pneumatic compression device (Sequential Circulator Model 2004; BioCompression Inc, Moonachie, NJ; Fig 2). Therapy sessions were performed for 1 hour twice daily (morning and evening) at 40 to 50 mm Hg while the subject was in a reclining or decubitus position. Compression therapy with IPC was performed over the compression bandage. In all cases, either the 19- or 31-in leg sleeves were used. Daily diaries were maintained by the study subjects, and the IPC devices and sleeves were checked every 4 weeks. In-service to the patient and family was provided. All subjects were followed weekly for 96 weeks. At each weekly evaluation visit, the wounds were measured, wound and pain assessments were performed, and adverse events (if any) were recorded. For most patients, bandage changes were performed twice weekly (once at the Wound Center and once by the visiting nurse). Wound measurements were performed using a 3-megapixel digital camera and photo-digital planimetry software (Pictzar; CDM BioVisual Inc, Elmwood Park, NJ).

## RESULTS

**Figure 3:**
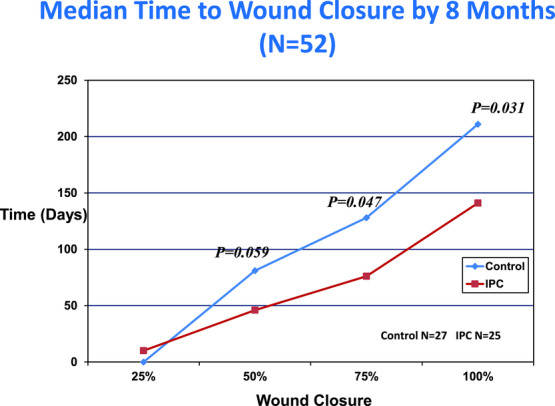
Median time to wound closure by 8 months. IPC indicates intermittent pneumatic compression.

**Figure 4:**
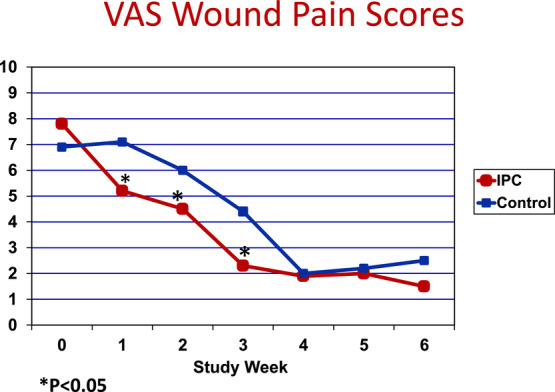
Effect of IPC therapy on local (wound) pain. IPC indicates intermittent pneumatic compression; VAS, visual analog scale.

There were no significant differences between the control and IPC treatment groups in patient demographics and baseline ulcer size and duration. The median time to wound closure at 8 months is shown in Figure 3. When compared with control treatment at the 8-month time point, IPC therapy reduced by 1.6-fold the median time to complete healing (*P* = .031). The rate of healing for both treatment groups is shown in Table 1. The speed of healing (in mm/d) was more than 2 times greater in the group receiving both standard compression bandages and IPC therapy (*P* = .41). The effect of IPC therapy on leg edema is shown in Table 2. After 20 weeks, the percent reduction in ankle and calf circumference was slightly greater favoring the IPC group, but this difference was not statistically significant. Local wound VAS pain scores for both treatment groups are shown in Figure 4. Significant (*P* < .05) wound pain relief was reported by study subjects receiving IPC only during the first 3 weeks of treatment. Thereafter, both treatment groups reported less wound pain.

## CONCLUSIONS

- The median time to healing by 9 months was 141 days for the IPC-treated group and 211 days for the control group (*P* = .031).

The rate of healing was 1.1 mm/d for the control group and 2.3 mm/d for the group treated with IPC (*P* < .05).

- Compared with subjects treated with compression alone, the group treated with IPC reported less pain at each evaluation point for the first 6 weeks.
- The IPC-treated group had greater reduction in leg edema (19% vs 11%), but this difference was not statistically significant.

## Figures and Tables

**Table 1:** Rate of healing*

Treatment	Control	IPC	*P*
Rate of closure (mm/day), mean ± SEM	0.8 ± 0.2	1.7 ± 0.5	.041
N	25	27	

*IPC indicates intermittent pneumatic compression; SEM, standard error of mean.

**Table 2:** *Leg edema*: Ankle and calf circumference*

Group	Ankle/calf, cm		
	Baseline	Week 20	% Δ
IPC plus 4-LB	46.5/59.3	37.6/48.2	19.1/18.7
4-LB (control)	44.7/56.4	39.6/49.2	12.0/13.2

*IPC indicates intermittent pneumatic compression; 4-LB, 4-layer bandage.
